# Comparison of Dexmedetomidine and Remifentanil on Adropin Expression in Unilateral Lumbar Microdiscectomy: A Prospective Active Controlled Randomized Trial Study

**DOI:** 10.3390/jcm14113711

**Published:** 2025-05-26

**Authors:** Gülay Gülbol-Duran, Senem Urfalı, Boran Urfalı

**Affiliations:** 1 Department of Medical Biology, Tayfur Ata Sokmen Faculty of Medicine, Hatay Mustafa Kemal University, 31060 Antakya, Hatay, Türkiye; 2 Department of Anesthesiology and Reanimation, Tayfur Ata Sokmen Faculty of Medicine, Hatay Mustafa Kemal University, 31060 Antakya, Hatay, Türkiye; senemurfali@mku.edu.tr; 3 Department of Neurosurgery, Tayfur Ata Sokmen Faculty of Medicine, Hatay Mustafa Kemal University, 31060 Antakya, Hatay, Türkiye; burfali@mku.edu.tr

**Keywords:** remifentanil, dexmedetomidine, adropin, eNOS, visual analogue scale (VAS), unilateral single-level lumbar microdiscectomy

## Abstract

**Background/Objectives:** Remifentanil and dexmedetomidine are widely used agents for pain management during general anesthesia. Adropin acts as a regulator of endothelial function by affecting nitric oxide bioavailability and various hemodynamic factors, including blood flow, vascular dilatation, and mean arterial pressure. We aimed to evaluate the effects of remifentanil and dexmedetomidine on adropin and eNOS levels and hemodynamic parameters in patients undergoing unilateral single-level lumbar microdiscectomy under controlled hypotension. **Methods:** This study included 40 patients who underwent lumbar microdiscectomy and were randomly assigned to two groups: 20 patients received remifentanil, and 20 received dexmedetomidine. Hemodynamic parameters, preoperative and postoperative VAS scores, and intraoperative blood loss were recorded. Adropin and eNOS mRNA levels were measured with RT-qPCR at three time points: preoperative (T1), intraoperative (T2), and postoperative (T3). Adropin protein levels were evaluated using ELISA. **Results:** The remifentanil and dexmedetomidine groups had similar heart rate, arterial pressure, intraoperative blood loss, surgery time, and VAS scores. The extubation time was longer with remifentanil. Adropin mRNA level was higher in remifentanil at all time points. At T2, the eNOS mRNA level was higher in the remifentanil group. In the dexmedetomidine group, adropin mRNA levels decreased at T2 compared to T1. Adropin protein levels were higher in the remifentanil group at T2 and T3. In the dexmedetomidine group, serum adropin levels decreased at T3 compared to those at T1. Preoperative VAS scores in patients receiving both remifentanil and dexmedetomidine were higher than postoperative VAS scores. No significant correlation was observed between VAS scores and adropin levels or between intraoperative blood loss and adropin protein levels. **Conclusions:** Both drugs demonstrated similar effects on the hemodynamics of the patients, and adropin levels were not associated with the VAS score and intraoperative blood loss. These findings suggest that dexmedetomidine mediates vasodilation through adropin-independent mechanisms, while remifentanil may provide more favorable surgical conditions through adropin in patients undergoing unilateral single-level lumbar microdiscectomy.

## 1. Introduction

Lumbar disc herniation (LDH) is a frequent and early indicator of lumbar spine degeneration, with an annual incidence of approximately 15 cases per 1000 adults. The reported incidence of LDH ranges from 2% to 3%, while its prevalence is estimated to be around 12% [1,2]. LDH presents with symptoms such as lower back pain, numbness in the lower extremities, and reduced mobility, significantly impacting the patient’s quality of life. Surgical intervention is recommended for patients who do not respond to conservative treatments, have severe conditions, or exhibit neurological deficits [3].

Adropin is a regulatory peptide, encoded by the energy homeostasis associated (ENHO) gene, playing a key role in metabolic cardiovascular homeostasis [4,5,6]. It is produced in the central nervous system (CNS) and peripheral organs, such as the heart, kidney, liver, pancreas, and human umbilical vein [5,6,7,8]. Beyond its role in glucose and lipid metabolism, adropin influences endothelial nitric oxide synthase (eNOS) activity, contributing to vascular function and nitric oxide (NO) bioavailability [5,9]. Adropin is also considered to influence cardiovascular dysfunction-related diseases. Endothelial cells exposed to adropin increase eNOS expression, which is responsible for nitric oxide (NO) bioavailability [8]. Adropin protein levels in the peripheral vasculature gradually decrease with age, whereas adropin supply has been shown to restore aging-related vasodilator dysfunction [10].

NO is an anti-atherosclerotic, anti-inflammatory, and anti-thrombotic molecule produced in most cells, where it promotes angiogenesis and reparative vasculogenesis [5,11]. When administered through the respiratory tract, NO can easily penetrate smooth muscle cells by transiting the alveolar epithelial cell barrier and stimulating cyclic guanosine monophosphate (cGMP)-mediated smooth muscle relaxation [11]. One of the endothelial protective functions of adropin is the regulation of NO bioavailability [10]. Previous studies have also reported that NO increases analgesic activity in several clinical settings, and NO levels are negatively correlated with the visual analog scale (VAS) scores of patients [12,13,14].

Remifentanil is a synthetic opioid analgesic with an ultra-short-acting pharmacokinetic profile, making it a preferred agent in various surgical procedures due to its rapid onset and easy dose adjustment [15]. Its immediate effects allow for precise intraoperative control and facilitate faster postoperative recovery [16]. However, its use may lead to hypotension, bradycardia, and postoperative secondary hyperalgesia due to increased opioid requirements [17,18].

Dexmedetomidine is a highly selective alpha-2 adrenergic receptor agonist with sedative, anxiolytic, sympatholytic, and analgesic properties, causing minimal respiratory depression [19]. After infusion, dexmedetomidine is rapidly distributed and primarily metabolized in the liver through glucuronidation and hydroxylation into inactive metabolites [20]. The major hemodynamic side effects of dexmedetomidine include hypotension, hypertension, and bradycardia, which result from its peripheral vasoconstrictive and sympatholytic effects [20].

eNOS-mediated endothelial function can be regulated by a prominent vasodilator called adropin, which modulates hemodynamic parameters, such as vascular dilation, mean arterial pressure, and blood flow [8,21]. The effects of the selected drugs on adropin expression remain unknown. The fact that adropin expression may change with a drug/drug combination will provide an idea of whether an adropin-mediated mechanism is used in vasodilation and blood flow. Hence, it will be possible to observe whether adropin expression affects the cleansing of the wound area and keeps the hemodynamic parameters within the desired optimal range during the surgical procedure.

This study aimed to investigate the effects of remifentanil and dexmedetomidine on adropin and eNOS expression in patients undergoing unilateral single-level lumbar microdiscectomy. To the best of our knowledge, this is the first study to examine the association between adropin expression and hemodynamic parameters in patients receiving remifentanil or dexmedetomidine.

## 2. Materials and Methods

### 2.1. Participants and Ethical Statement

The current study was conducted at Hatay Mustafa Kemal University following ethical approval from the Clinical Research Ethics Committee (Date-No: 04/06/2020-16). All experimental procedures involving human participants were conducted in accordance with the Declaration of Helsinki. Candidates who applied to the Neurosurgery Polyclinic at Hatay Mustafa Kemal University Research and Training Hospital between July 2020 and July 2022 and met the inclusion criteria were interviewed in their hospital rooms prior to surgery. After the surgeon provided a comprehensive explanation of the study, both verbal and written consent were obtained from the participants.

The inclusion criteria for this study were as follows: patients aged over 18 years, classified as American Society of Anesthesiologists (ASA) I-III [22], undergoing surgery for LDH for the first time with unilateral, single-level lumbar disc herniation presenting with both clinical symptoms and radiological evidence. All patients had a positive straight leg raise test, with results ranging between 30° and 60°, and their magnetic resonance imaging (MRI) or computed tomography findings were correlated with their symptoms.

**Figure 1:**
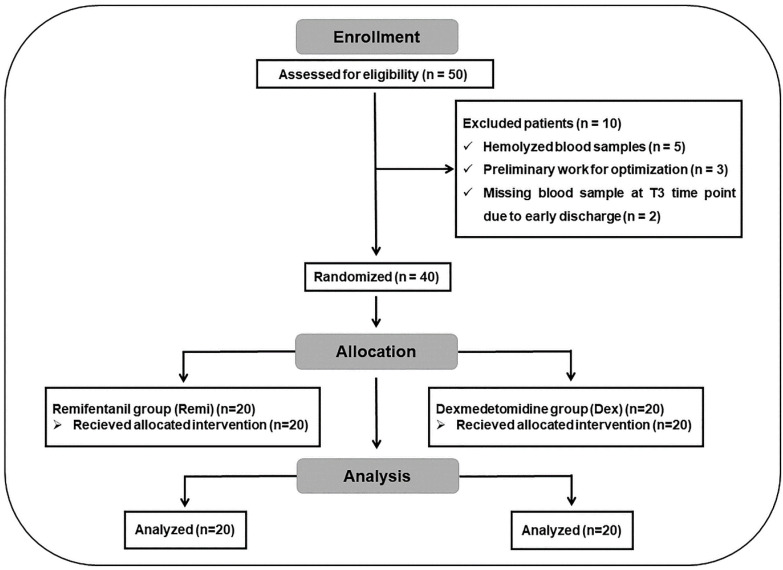
CONSORT flow diagram of the study.

Patients with mental disorders, cardiac arrhythmia, Mobitz type-2 heart block, coronary artery disease, ischemic heart disease, liver or kidney diseases, known hypersensitivity or allergic reactions to the study drugs, or those who had undergone revision surgery for recurrent LDH were excluded from the study. Based on a clinical effect size of 0.2 (Cohen’s d) between the relative mean mRNA values of the remifentanil and dexmedetomidine groups, a sample size calculation was performed with a maximum type I error rate of 5% and a minimum statistical power of 90%. As a result, 20 patients were included in each group, for a total of 40 patients who underwent single-level unilateral lumbar microdiscectomy. The total number of patients was determined using power analysis (G-power version 3.1.9.6). Before the study, a randomization table was created using the random matching method E-PICOS AI Smart Biostatistics Software (version 21.3, New York, NY, USA), and patient selection was performed using the double-blind method. The CONSORT flow diagram of the study is presented in Figure 1. The initial target sample size was 50 patients; however, following the exclusion of 10 patients, the final randomized sample size for each group was 20.

### 2.2. Anesthesia Protocols

In the operating room, standard monitoring was applied, including lead II electrocardiography, pulse oximetry, and noninvasive blood pressure measurement. Following preoxygenation with 100% oxygen, general anesthesia was induced with lidocaine (1 mg/kg), fentanyl (2 µg/kg), propofol (2 mg/kg), and 0.6 mg/kg rocuronium bromide (0.6 mg/kg). Endotracheal intubation was performed after confirming adequate muscle relaxation. Anesthesia was maintained with 2% sevoflurane in a 50:50 air/oxygen mixture. If required, an additional intravenous dose of rocuronium bromide (0.5 mg/kg) was administered intraoperatively to maintain the neuromuscular blockade.

Following intubation, the patients were positioned prone on the operating table, and a continuous infusion of remifentanil (0.1–0.2 μg/kg/min without a loading dose; Rentanil 2 mg, Vem İlaç, Istanbul, Türkiye) or dexmedetomidine (1 μg/kg loading dose over 10 min, followed by 0.2–0.7 μg/kg/h maintenance infusion; Dekstomid 200 mcg/2 mL, Polifarma, Tekirdağ, Türkiye) was administered throughout the surgical procedure. To optimize the surgical field and reduce intraoperative bleeding, controlled hypotension was applied during surgery, with a target mean arterial pressure (MAP) of 60–65 mmHg. Anesthetic agents were titrated accordingly to achieve and sustain this level of arterial pressure. The depth of anesthesia and neuromuscular blockade were monitored using clinical parameters, including hemodynamic responses, respiratory patterns, and physical reactions to surgical stimuli.

Anesthetic agents were discontinued at the onset of skin suturing, and any residual neuromuscular blockade was reversed with 4 mg/kg of sugammadex sodium. Patients were extubated once they met the criteria for tracheal extubation, which included a respiratory rate >10 breaths/min, spontaneous breathing with a tidal volume of ≥10 mL/kg, and the ability to maintain adequate neuromuscular functions, such as sustaining an arm lift. Following surgery, all patients were transferred from the operating room to the post-anesthesia care unit (PACU). Patients were discharged from the PACU once they reached a modified Aldrete score of ≥9 and had adequate motor function in the operated leg [23].

### 2.3. Surgical Protocols

All surgeries were performed by a senior neurosurgeon. Following meticulous preparation, the surgical team proceeded with the surgery. C-arm fluoroscopy (Ziehm Solo Mobile C-Arm; Ziehm Imaging GMBH, Nürnberg, Germany) was used to accurately identify the target level.

After identifying the target level, microdiscectomy was performed using a neurosurgical microscope (Zeiss OPMI Vario/NC 33 System; Carl Zeiss AG, Oberkochen, Baden-Württemberg, Germany).

The surgical procedure was completed with hemostasis and proper layer closures.

The surgical protocol was performed using hypotensive anesthesia. There are two main reasons why the patient should be operated on under controlled hypotensive anesthesia. The first is systemic effects, which include possible hemodynamic deterioration due to bleeding and negative effects on the entire body and neural tissue. The second important effect for neurosurgical practice is that if the patient is not under controlled hypotension during this surgical procedure, which is performed in a very narrow area and in an area with almost no dead space anatomically, the bleeding that may occur will affect both the surgical field of vision and clarity and will cause complications such as neurological deficits by pressing on the spinal cord, spinal roots, and epidural bleeding. [24].

Surgery and extubation time (min) and intraoperative blood loss (mL) were recorded during the procedure, and heart rate (beats/min) and systolic and diastolic blood pressures (mm Hg) were measured at predefined time points (induction, 5th, 15th, 30th, 45th, 60th, 90th, and 120th min). Preoperative (pre-op) and postoperative (post-op) pain levels in both the remifentanil and dexmedetomidine groups were measured using a 10-point VAS [25]. VAS scores were recorded pre-op and post-op (24 h after surgery when the mobility of the patient was confirmed).

### 2.4. Blood Sample Collection

Serum adropin protein levels were analyzed in patients from the treatment groups at three different time points: T1 (30 min before anesthesia induction; pre-op), T2 (45 min after surgery onset; intra-op), and T3 (24 h postoperatively; post-op). For this purpose, 5 mL peripheral blood samples were collected in ethylenediaminetetraacetic acid (EDTA)-free tubes. Relative mRNA levels of eNOS and adropin were also measured using 2 mL peripheral blood samples collected from all patients in EDTA-containing tubes. For gene expression analyses of adropin and eNOS, 300 µL blood was mixed with 700 µL RNA stabilizer agent (RNA save, Biological Industries, Göttingen, Germany) and stored at −80 °C until the day of analyses.

### 2.5. Enzyme-Linked Immunosorbent Assay (ELISA) Analyses

The blood samples in EDTA-free tubes were centrifuged at 2000× *g* for 10 min after 20–30 min of incubation at room temperature. The isolated serum samples were stored at −80 °C until further experiments. Serum adropin levels were measured using a commercial ELISA kit (Bioassay Technology Laboratory, E3231Hu, Jiaxing, Zhejiang, China). The intra- and inter-assay coefficient of variation values for adropin were 2.98–4.56% and <10%.

### 2.6. Relative mRNA Expression Analysis

**Figure 2:**
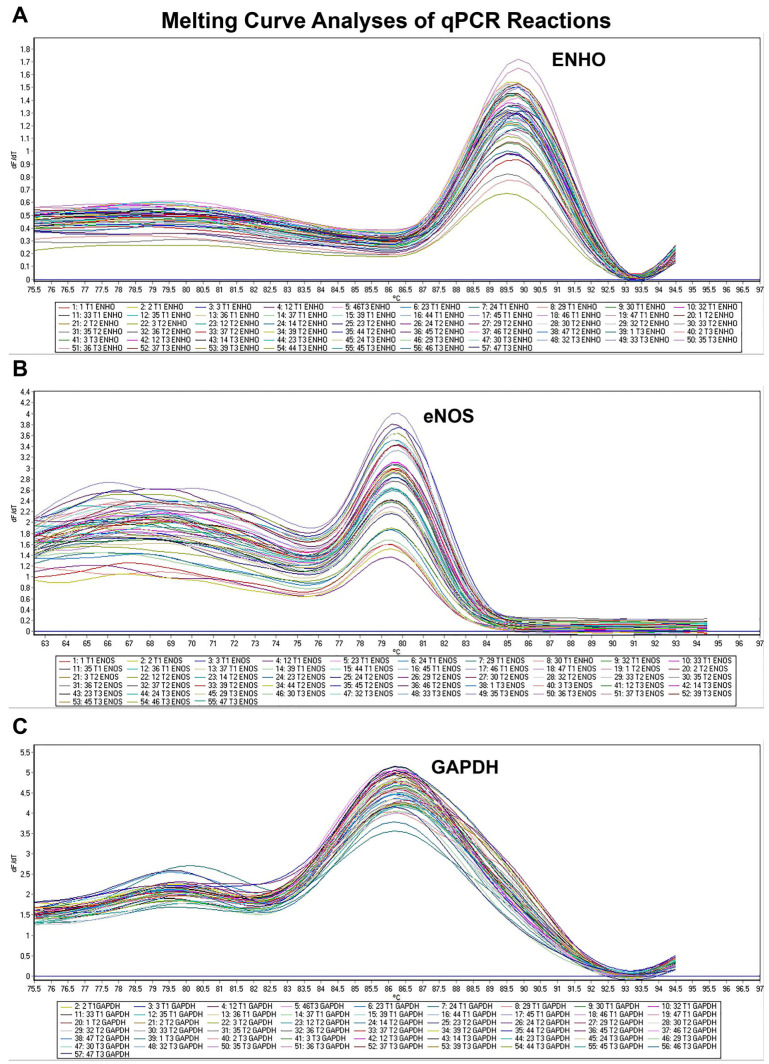
Melting curve analyses after qPCR reactions for ENHO (**A**), eNOS (**B**), and GAPDH (**C**) genes. ENHO, energy homeostasis associated (adropin); eNOS, nitric oxide synthase 3; GAPDH: Glyceraldehyde-3-phosphate dehydrogenase.

Total RNA was isolated using a commercial kit (Hibrigen, MG-RNA-01, Kocaeli, Türkiye) from whole blood samples. After measuring RNA concentrations using a spectrophotometer (Thermo Scientific Multiscan Go, Vantaa, Finland), RNA samples were pre-diluted for further cDNA synthesis (abm, OneScript Plus, Vancouver, BC, Canada) with the following reaction conditions in a thermal cycler (BioRad, Hercules, CA, USA): 15 min at 50 °C, 5 min at 85 °C, and storaed at 4 °C. cDNAs were pre-diluted (1/5) for further qPCR reactions (HOT FIREPol^®^ EvaGreen^®^ qPCR Mix Plus, Solis Biodyne, Tartu, Estonia) using RotorGene Q (Qiagen, San Diego, CA, USA) under the following conditions: initial denaturation for 12 min at 95 °C; 40 cycles of 15 s at 95 °C, 40 s at 65 °C, and 40 s at 72 °C; final melting curve analysis between 61 °C and 95 °C. The specific primers used in the qPCR reactions for adropin (ENHO), eNOS, and GAPDH are listed in Table 1, and the melting curve analyses are depicted in Figure 2. The raw cycle threshold (Ct) values were normalized to the internal housekeeping control GAPDH, and the relative mRNA levels of the target genes were calculated using the 2^−ΔCt^ method [26].

### 2.7. Statistical Analysis

All statistical analyses and plots were prepared using GraphPad software (version 8.0.2, Boston, MA, USA). Categorical values in two independent groups were compared using Fisher’s exact test and expressed as percentages. Gaussian distribution of non-categorical data was tested using the Shapiro–Wilk test. For the comparison of parametric paired values in more than two groups, repeated measures one-way ANOVA was used with post hoc Holm–Sidak’s multiple comparisons test. Non-parametric paired values in more than two groups were compared using the Friedman test with Dunn’s multiple comparisons test. For comparison of parametric and non-parametric values in two independent groups, independent *t*-test and Mann–Whitney U test were used, respectively. Data are expressed as percentages for categorical variables and as medians (25–75% quartiles). The *p*-values less than 0.05 were considered statistically significant.

## 3. Results

### 3.1. Demographic and Clinical Parameters of Subjects

A total of forty patients were included in this prospective clinical study. The demographic and clinical parameters of the participants are summarized in Table 2. There were no significant differences between the two groups regarding age, sex, or body mass index (BMI) (*p* ≥ 0.05).

Hypertension and diabetes mellitus were the most common comorbidities in both groups. In the remifentanil group, hypertension was observed in four patients (20%) and diabetes mellitus in four patients (20%). Additionally, benign prostatic hyperplasia and previous thyroid surgery were present in one patient (5%). In the dexmedetomidine group, hypertension was noted in three patients (15%), diabetes mellitus in two patients (10%), and chronic obstructive pulmonary disease in one patient (5%).

**Figure 3:**
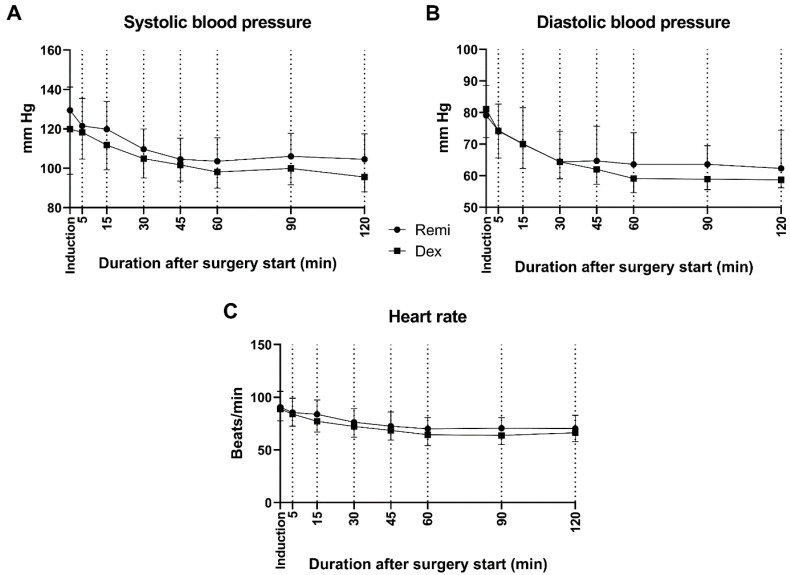
Intraoperative systolic (**A**) and diastolic (**B**) blood pressures and heart rates (**C**) of patients in the remifentanil and dexmedetomidine groups. Remi: Remifentanil, Dex: Dexmedetomidine.

No significant differences were observed between the groups in terms of surgery duration, intraoperative blood loss, heart rate, or systolic and diastolic blood pressures (Table 2, Figure 3, *p* ≥ 0.05). However, extubation time was significantly longer in the remifentanil group than in the dexmedetomidine group (*p* < 0.05). In the dexmedetomidine and remifentanil groups, the post-op VAS, heart rate, and systolic and diastolic blood pressure values of the patients significantly decreased compared to the pre-op values (Table 2, *p* < 0.05). On the other hand, there was no significant difference between the post-op VAS scores of dexmedetomidine and remifentanil-treated patients (Table 2, *p* ≥ 0.05).

### 3.2. Adropin and eNOS Expression Levels

**Figure 4:**
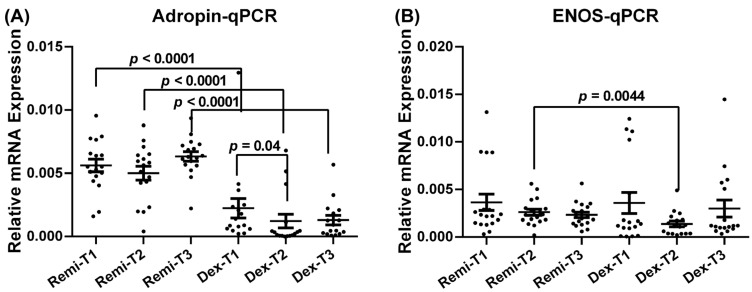
Relative mRNA expression levels of adropin (**A**) and eNOS (**B**) of the patients at T1, T2, and T3 time points in the remifentanil and dexmedetomidine groups. The difference between the same time points in the remifentanil and dexmedetomidine groups was tested using an unpaired *t*-test, while the difference between time points in the same groups was tested using the Friedman test with Dunn’s multiple comparisons test. Data are presented as mean ± SEM. *p* < 0.05 was considered statistically significant. T1: 30 min pre-anesthesia, T2:45 min after surgery initiation, T3:24 h after surgery. Remi: Remifentanil, Dex: Dexmedetomidine. eNOS: Endothelial nitric oxide synthase.

Adropin and eNOS mRNA expression levels in the two anesthetic groups (remifentanil and dexmedetomidine), analyzed with the qPCR method, are presented in Figure 4. In each group, the target parameters were measured at three different time points (T1, T2, and T3) to evaluate the effect of remifentanil and dexmedetomidine on the expression pattern of the target parameters.

Patients who received remifentanil exhibited significantly higher adropin mRNA levels at T1, T2, and T3 than those who received dexmedetomidine (Figure 4A, *p* < 0.05). Additionally, in the dexmedetomidine group, adropin mRNA levels at T1 were significantly higher than those at T2 (Figure 4A, *p* < 0.05). The eNOS mRNA level at T2 was also significantly higher in the remifentanil group than in the dexmedetomidine group (Figure 4B; *p* < 0.05).

**Figure 5:**
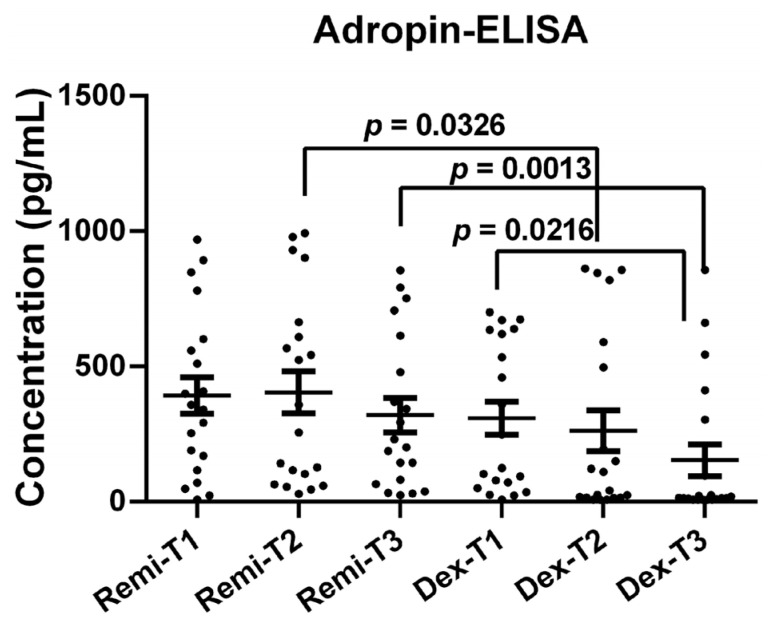
Adropin protein levels of the patients at T1, T2, and T3 time points in the remifentanil and dexmedetomidine groups. The difference between the same time points in the remifentanil and dexmedetomidine groups was tested with an unpaired *t*-test, while the difference between time points in the same groups was tested with the Friedman test with Dunn’s multiple comparisons test. Data were presented as mean ± SEM. *p* < 0.05 was considered statistically significant. T1: 30 min pre-anesthesia, T2: 45 min after surgery starts, T3: 24 h after surgery. Remi: Remifentanil, Dex: Dexmedetomidine.

Adropin protein levels were also measured using the ELISA method after detecting significant alterations in adropin mRNA levels at each time point in the remifentanil and dexmedetomidine groups. Adropin protein levels at T2 and T3 were significantly higher in patients receiving remifentanil than in those receiving dexmedetomidine (Figure 5, *p* < 0.05).

Additionally, in the dexmedetomidine group, adropin protein levels at T3 were significantly lower than those at T1 (Figure 5, *p* < 0.05).

### 3.3. Correlation Analyses of Serum Adropin Levels with Clinical Parameters

**Figure 6:**
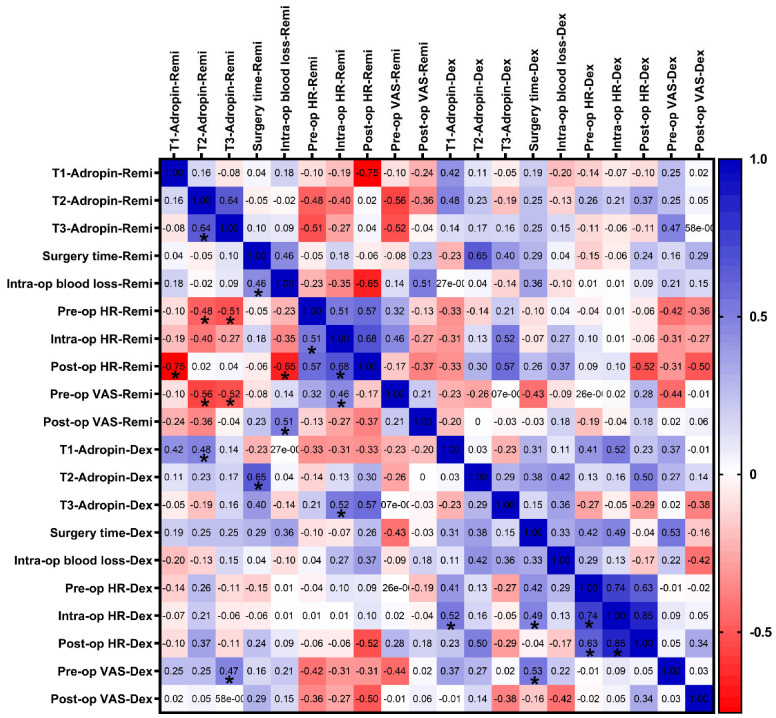
Correlation analysis between clinical parameters and adropin protein levels in the remifentanil and dexmedetomidine groups. A correlation matrix was formed based on Spearman’s correlation and depicted as a two-color gradient heat map. Each cell contains the correlation coefficient. * *p* < 0.05. T1: 30 min pre-anesthesia, T2: 45 min after surgery starts, T3: 24 h after surgery. Remi, Remifentanil, Dex: Dexmedetomidine, VAS: Visual analog scale; pre-, intra-, and post-op, before operation, 45 min, and 120 min after operation starts; SBP: Systolic blood pressure; DBP: Diastolic blood pressure.

Correlation analyses were conducted to evaluate the potential associations between serum adropin levels and selected clinical and surgical parameters in both the remifentanil and dexmedetomidine groups (Figure 6). We found no significant correlation between the clinical parameters (e.g., pre-op and post-op VAS, intra-op blood loss, etc.) and adropin levels at the corresponding time point (i.e., T1 vs. pre-op, T2 vs. intra-op, or T3 vs. post-op).

## 4. Discussion

Adropin is a highly preserved 76-aminoacid-long peptide hormone which is encoded by the energy homeostasis associated (ENHO) gene [6]. In addition, adropin is linked to cardiovascular homeostasis as a regulator of endothelial function [8]. An accumulating number of studies have revealed that adropin possesses significant cardiovascular functions [21,27,28]. Adropin has been identified as a vasodilator that regulates endothelial function via eNOS activation [8,10,27]. As a regulator of endothelial function, adropin influences various hemodynamic factors, including blood flow, vascular dilation, and mean arterial pressure [21]. In this study, we aimed to investigate the effects of remifentanil and dexmedetomidine on adropin and endothelial nitric oxide synthase (eNOS) expression levels. We also aimed to determine how the vasodilator function of adropin is affected at each time point in the context of the vital importance of controlled hypotension during surgical operations. To the best of our knowledge, this is the first study to specifically examine these effects, highlighting the novelty of our work.

Previous studies have demonstrated a negative correlation between serum adropin levels and various diseases, highlighting the protective role of high adropin levels in conditions such as rheumatoid arthritis, diabetes, coronary atherosclerosis, and nonalcoholic fatty liver disease [28,29,30,31]. These studies emphasize the beneficial effects of adropin on vascular function and hemodynamic stability, as well as the relationship between the reduction of pain in the postoperative period.

Our findings further support the role of adropin in hemodynamic regulation, demonstrating that while adropin mRNA expression remained stable in the remifentanil group, it significantly decreased in the dexmedetomidine group during the intraoperative period. ELISA results confirmed a delayed but consistent reduction in adropin protein levels in the dexmedetomidine group at T3. In the dexmedetomidine group, the delayed decrease in adropin protein levels at T3 compared to the decrease in mRNA levels at T2 arises more likely from the fact that, physiologically, protein synthesis occurs later than mRNA synthesis in a cell.

Previous studies have reported that adropin exerts its vasodilatory effect via eNOS [8,30]. Adropin was also demonstrated to increase the bioavailability of NO in the body [5]. NO was previously shown to alleviate pain more effectively when administered with NSAIDs than with NSAIDs alone [14]. In addition, surgeries performed with additional inhaled NO provided better oxygenation and earlier discharge of the patients from the cardiac intensive care unit [31]. Another clinical study searched for an association between NO levels and pain during acute vaso-occlusive sickle cell crisis and found that initial NO levels in patients with persistent pain were higher than those in patients with alleviated pain [12]. In another noteworthy study, the combined effects of analgesics on NO levels were also investigated. It was observed that patients receiving dexmedetomidine-sufentanil exhibited lower NO levels and higher VAS scores than those receiving midazolam-sufentanil [13]. In this context, the gene expression levels of eNOS at the three time points in both groups were analyzed. We observed that at T2, the eNOS mRNA level was significantly higher in the remifentanil group than in the dexmedetomidine group. These outcomes suggest that remifentanil acts through adropin-promoted eNOS during controlled hypotension regulation, while dexmedetomidine downregulated adropin expression and consequently suppressed eNOS expression in the present study. These findings suggest that remifentanil may support controlled hypotension via the adropin-eNOS pathway, whereas dexmedetomidine likely induces vasodilation through alternative mechanisms.

Controlled hypotension is commonly used to minimize blood loss and improve the visibility of the surgical field [29,32]. Dexmedetomidine and remifentanil are frequently used for this purpose due to their fast-acting and precise arterial pressure control dexmedetomidine, an α_2_-adrenergic receptor agonist, reduces blood pressure by inhibiting norepinephrine secretion [33], while remifentanil, a μ-opioid receptor agonist, allows rapid recovery with stable hemodynamics [34,35]. Some studies reported superior hypotension control and shorter extubation times with remifentanil [36], while others found no significant differences in blood loss or hemodynamic parameters between the two agents [32,37]. Our study supports the findings showing no significant differences in hemodynamic effects between dexmedetomidine and remifentanil, although we observed longer extubation times in the remifentanil group, which contrasts with prior reports [36,38,39]. This difference may be explained by factors such as patient characteristics, dosage range administered, duration of anesthesia, or timing of drug discontinuation toward the end of the operation. These findings need to be confirmed in larger patient cohorts for a better understanding.

Postoperative pain management outcomes are also conflicting. While some studies suggest that dexmedetomidine provides superior pain relief in lumbar fusion surgery [37], others, including Naik et al., found no significant differences in postoperative VAS scores between dexmedetomidine and placebo [40]. Our results revealed that the post-op VAS scores of the same patients in the dexmedetomidine and remifentanil groups were significantly lower than the pre-op VAS values; however, there were no significant differences in post-op pain management between the dexmedetomidine and remifentanil groups.

Correlation analysis revealed no significant associations between serum adropin levels and clinical parameters, such as surgery duration, intraoperative blood loss, heart rate, and blood pressure. Furthermore, we analyzed the association between adropin protein levels and the pre-op and post-op VAS scores of the same patients in the dexmedetomidine and remifentanil groups and found no significant correlation at the corresponding time points.

However, our findings suggest that adropin may still play a role in hemodynamic regulation at the molecular level. Specifically, remifentanil maintained consistent adropin and eNOS expression, whereas dexmedetomidine downregulated adropin, potentially contributing to its hypotensive effect. Although our findings do not reveal a clear difference in the superiority of these two drugs in clinical practice, they provide new insights into the mechanisms underlying controlled hypotension and highlight potential differences in the vascular effects of these anesthetic agents.

### Study Limitations

The primary limitations of this study include the small sample size and single-center design, which may affect the generalizability of the findings. The lack of eNOS protein quantification via ELISA could have provided a more comprehensive understanding of adropin-mediated vascular regulation. Furthermore, this study focused on intraoperative hemodynamic effects without long-term follow-up, limiting conclusions regarding sustained hemodynamic and vascular outcomes. Finally, the lack of a mechanistic approach to the major findings with additional in vitro and/or in vivo experimental disease models limits the understanding of the underlying mechanism of adropin in pain regulation and endothelial function. Future studies with larger cohorts, extended biochemical analyses, and mechanistic study designs are necessary to validate and expand these findings.

## 5. Conclusions

Both remifentanil and dexmedetomidine had comparable effects on the hemodynamics and pain management in patients who underwent unilateral single-level microdiscectomy. However, the reduction in adropin levels in the dexmedetomidine group suggests that dexmedetomidine induces vasodilation through alternative pathways. Given adropin’s vascular role, our findings suggest that remifentanil may be more favorable for maintaining hemodynamic balance during lumbar spine surgery. We also found no evident association between the VAS scores and adropin levels. Further studies are needed to confirm these findings.

## Figures and Tables

**Table 1:** qPCR primer sequences.

Gene Symbol	Primer Sequence	Amplicon (bp)	Anneal. (°C), Cycle	Ref. Seq.
ENHO	F: 5′-CTCAACTCAGGCTCAGGACT-3′ R: 5′-GACAGTGGAGCTGCCTCAAT-3′	144	65, 40×	NM_198573.3
eNOS	F: 5′-GAAGGCGACAATCCTGTATGGC-3′ R: 5′-TGTTCGAGGGACACCACGTCAT-3′	135	65, 40×	NM_000603.5
GAPDH	F: 5′-GTCTCCTCTGACTTCAACAGCG-3′ R: 5′- ACCACCCTGTTGCTGTAGCCAA -3′	131	65, 40×	NM_002046.7

ENHO, energy homeostasis associated; eNOS, endothelial nitric oxide synthase; GAPDH: Glyceraldehyde-3-phosphate dehydrogenase; F, forward primer sequence; R, reverse primer sequence; bp, base pair; Anneal: Annealing temperature; X, times of cycle, Ref. Seq.: NCBI reference sequence.

**Table 2:** Demographic and clinical parameters of the participants.

	Remi (n = 20)	Dex (n = 20)	*p*-Value
Age, year	46.3 (12.6)	42.4 (10.9)	0.295 ^a^
Sex			0.320 ^b^
Female	11/20 (55%)	15/20 (75%)	
Male	9/20 (45%)	5/20 (25%)	
BMI, kg/m^2^	24.8 (2.2)	24.1 (2.4)	0.405 ^a^
Diseases			
BPH	1/20 (5%)	-	
Thyroid surgery	1/20 (5%)	-	
HT	4/20 (20%)	3/20 (15%)	
DM	4/20 (20%)	2/20 (10%)	
COPD	-	1/20 (5%)	
Surgery time, min	100.0 (77.5–120.0)	120.0 (96.3–120.0)	0.468 ^c^
Extubation time, min	9.0 (7.3–10.0)	6.0 (3.0–9.3)	**0.002** ^c^
Intra-op blood loss, mL	120.0 (80.0–150.0)	110.0 (62.5–187.5)	0.929 ^c^
VAS			
Pre-op VAS	9.0 (8.3–9.8)	9.5 (9.0–10.0)	0.198 ^c^
Post-op VAS	1.5 (0.3–3.0)	1.00 (1.0–2.0)	0.548 ^c^
*p*-value	**<0.0001** ^d^	**<0.0001** ^d^	
Heart rate, beats/min			
Pre-op	90.0 (78.5–100.8)	91.5 (79.8–98.0)	0.680 ^a^
Intra-op	75.0 (59.8–80.8)	69.0 (60.0–77.0)	0.287 ^a^
Post-op	69.5 (58.8–85.0)	65.5 (59.0–74.0)	0.377 ^a^
*p*-value	**<0.0001** ^e1^ **0.0052** ^e2^ 0.804 ^e3^	**<0.0001** ^e1^ **<0.0001** ^e2^ 0.456 ^e3^	
SBP, mm Hg			
Pre-op	128.5 (120.0–140.0)	127.5 (111.3–135.8)	0.249 ^c^
Intra-op	103.0 (96.3–111.5)	100.0 (94.8–106.8)	0.343 ^a^
Post-op	102.0 (92.5–114.0)	96.0 (90.0–99.0)	0.093 ^c^
*p*-value	**<0.0001** ^e1^ **0.0006** ^e2^ 0.999 ^e3^	**0.011** ^e1^ **0.009** ^e2^ **0.010** ^e3^	
DBP, mm Hg			
Pre-op	80.0 (74.3–84.0)	81.0 (75.0–88.0)	0.511 ^a^
Intra-op	69.5 (53.0–74.0)	63.0 (58.0–65.8)	0.343 ^c^
Post-op	60.0 (51.0–76.0)	59.0 (56.3–60.0)	0.316 ^a^
*p*-value	**<0.0001** ^e1^ **0.002** ^e2^ 0.788 ^e3^	**<0.0001** ^e1^ **<0.0001** ^e2^ **0.011** ^e3^	

Data are presented as mean (standard deviation), median [interquartile range], or n/total N (%). Remi: Remifentanil, Dex: Dexmedetomidine, BMI: Body mass index, BPH: Benign prostatic hyperplasia, HT: Hypertension, DM: Diabetes mellitus, COPD: Chronic obstructive pulmonary disease, VAS: Visual analog scale, pre-, intra- and post-op: Before operation, 45 min and 120 min after operation starts, SBP: Systolic blood pressure, DBP: Diastolic blood pressure. a: Unpaired *t*-test, b: Fisher’s exact test, c: Mann–Whitney U test, d: Wilcoxon matched-pairs signed rank test, e: Mixed-effects model (REML: restricted maximum likelihood)—Tukey’s multiple comparisons test, e1: Pre-op vs. Intra-op, e2: Pre-op vs. Post-op, e3: Intra-op vs. Post-op.

## Data Availability

The raw data supporting the conclusions of this article will be made available anonymously by the authors upon request due to patient privacy concerns.

## Abbreviations

ASA	American Society of Anesthesiologists
BMI	Body Mass Index
BPH	Benign prostatic hyperplasia
cGMP	Cyclic guanosine monophosphate
CNS	Central nervous system
COPD	Chronic obstructive pulmonary disease
Ct	Cycle threshold
Dex	Dexmedetomidine
DM	Diabetes mellitus
EDTA	Ethylenediaminetetraacetic acid
ELISA	Enzyme-Linked ImmunoSorbent Assay
ENHO	Energy homeostasis associated
eNOS	Endothelial nitric oxide synthase
GAPDH	Glyceraldehyde-3-phosphate dehydrogenase
HT	Hypertension
LDH	Lumbar disc herniation
MAP	Mean arterial pressure
MRI	Magnetic resonance imaging
NSAID	Non-steroid anti-inflamatory drug
NO	Nitric oxide
PACU	Post-anesthesia care unit
pre-op	Preoperative
post-op	Postoperative
Remi	Remifentanil
RT-qPCR	Quantitative reverse transcription polymerase chain reaktion
SBP	Systolic blood pressure
VAS	Visual analogue scale
